# Chemical Biology Meets Metabolomics: The Response of Barley Seedlings to 3,5-Dichloroanthranilic Acid, a Resistance Inducer

**DOI:** 10.3390/molecules30030545

**Published:** 2025-01-25

**Authors:** Claude Y. Hamany Djande, Paul A. Steenkamp, Ian A. Dubery

**Affiliations:** Research Centre for Plant Metabolomics, Department of Biochemistry, University of Johannesburg, P.O. Box 524, Auckland Park, Johannesburg 2006, South Africa; claudeh@uj.ac.za (C.Y.H.D.); psteenkamp@uj.ac.za (P.A.S.)

**Keywords:** barley, chemical biology, induced disease resistance, metabolomics, priming, stress response, 3,5-dichloroanthranilic acid

## Abstract

Advances in combinatorial synthesis and high-throughput screening methods have led to renewed interest in synthetic plant immunity activators as well as priming agents. 3,5-Dichloroanthranilic acid (3,5-DCAA) is a derivative of anthranilic acid that has shown potency in activating defence mechanisms in Arabidopsis and barley. Chemical biology, which is the interface of chemistry and biology, can make use of metabolomic approaches and tools to better understand molecular mechanisms operating in complex biological systems. Here we report on the untargeted metabolomic profiling of barley seedlings treated with 3,5-DCAA to gain deeper insights into the mechanism of action of this resistance inducer. Histochemical analysis revealed the production of reactive oxygen species in the leaves upon 3,5-DCAA infiltration. Subsequently, methanolic extracts from different time periods (12, 24, and 36 h post-treatment) were analysed by ultra-high-performance liquid chromatography hyphenated to a high-resolution mass spectrometer. Both unsupervised and supervised chemometric methods were used to reveal hidden patterns and highlight metabolite variables associated with the treatment. Based on the metabolites identified, both the phenylpropanoid and octadecanoid pathways appear to be main routes activated by 3,5-DCAA. Different classes of responsive metabolites were annotated with flavonoids, more specifically flavones, which were the most dominant. Given the limited understanding of this inducer, this study offers a metabolomic analysis of the response triggered by its foliar application in barley. This additional insight could help make informed decisions for the development of more effective strategies for crop protection and improvement, ultimately contributing to crop resilience and agricultural sustainability.

## 1. Introduction

Food security and sustainable agriculture are central themes within the ‘zero hunger’ sustainable development goal set by the United Nations in 2015. Emerging threats, such as climate change disrupting agricultural productivity, add layers of complexity to the task of preserving food security [1]. Plants use diverse defence mechanisms (constitutive or induced) to overcome the biotic and abiotic factors interfering with their growth and yield [2]. Induced defence mechanisms are often initiated by the recognition of conserved pathogen-associated molecular patterns (PAMPs) and result in the establishment of pattern-triggered immunity (PTI) and a broad spectrum of associated defence mechanisms against a range of environmental stresses [3,4]. This enhanced resistance can be prompted by an initial localised pathogen infection or by elicitors or inducers mimicking the pathogen invasion, causing the biological activation of induced defence mechanisms [5,6,7].

The concept of elicitors dates back to the 1970s, when some microbial molecules were found to stimulate the production of antimicrobial small-molecule phytoalexins in plants. The external application of elicitors to enhance plant immunity (i.e., induced resistance) has been a promising strategy in agricultural science, bolstering plant resilience to a range of stressors that include broad-spectrum resistance against disease. Such exposure can activate intricate signalling cascades in plants, leading to the up-regulation of defence-related genes, the fluctuation in the production of secondary metabolites, and the reinforcement of physical barriers against environmental stresses [8]. Relatedly, the perturbation occurring in the plant (e.g., priming) may result in a primed state of alertness that enables the plant to mount a faster, stronger, and more effective defence response upon secondary exposure to stress [5,9,10,11]. These elicitors may be molecular signatures found on (non)pathogenic microbes or chemicals of natural or synthetic origin [12,13]. Synthetic elicitors are drug-like compounds exhibiting structural differences compared to natural defence elicitors [14]. Targeted, transient activation of plant immunity by exogenously applied chemicals may be valuable in resisting virulent pathogens that threaten crops only during a limited growth period or under specific environmental conditions. However, the success of these approaches may be dependent on the uptake of the inducer and the triggered biochemical responses as determined by the plant’s unique genotype.

Chemical biology refers to research at the interface of chemistry and biology, where concepts and approaches from both disciplines are used to better understand molecular mechanisms operating in complex biological systems [6,14]. Through the application of small molecules, chemical biology allows for the precise manipulation of plant signalling pathways and the elucidation of complex molecular mechanisms underlying priming. This field leverages synthetic chemistry to design novel compounds that can mimic, enhance, or inhibit natural signalling molecules, thereby providing tools to study and improve plant resilience [15]. However, the challenge remains to pinpoint the target(s) of these bioactive chemicals to unveil the genes, proteins, or metabolic pathways responsible for a specific phenotype. The metabolome is regarded as a determinant of the phenotype, which in turn is the reflection of the upstream genotype. Chemical biology can thus be combined with other omics-based profiling technologies, particularly metabolomics [16,17,18].

Progress in combinatorial synthesis and high-throughput screening techniques has sparked a renewed interest in synthetic plant-immunity activators and priming agents [6,19]. The complexity of biological systems can be elucidated by identifying and quantifying key small primary and secondary metabolites. These metabolites serve as intermediates, signalling molecules, and energy carriers, collectively orchestrating the intricate biochemical reactions that allow plants to respond to environmental cues. Metabolomics is an established, rapidly evolving, and pioneering scientific tool used to investigate metabolites within an organism. As such, metabolomics is a powerful approach within systems biology that focuses on the comprehensive profiling of metabolites in biological systems. It potentially provides a snapshot of the cellular metabolic state and reflects the dynamic interplay of genetic, environmental, and physiological factors.

Barley (*Hordeum vulgare* L.) is a versatile cereal crop with a significant role in global agriculture. Its adaptability and diverse cultivated varieties (cvs) make it a valuable resource for both human consumption and animal feed [20,21]. Barley is subject to a number of pests and diseases, such as powdery mildew, leaf spot, rust, and blotch. Among these biotic stressors is *Pyrenophora teres* f. *teres*, the causal agent of the ‘net blotch net type’ disease, which has caused significant crop failures or yield losses [22,23]. Inducible metabolic responses in support of natural defences are of great importance, as they play a pivotal role in crop yield, quality, and overall resilience. These synthetic chemical inducers are designed to mitigate the adverse effects of abiotic and biotic environmental stressors. Rather than having a direct antimicrobial effect on pathogens, inducers of plant immunity may activate, strengthen, or prime defence capabilities [24,25]. By enhancing the plant’s focused metabolic responses, this augmented resilience can lead to increased crop productivity and quality. Dichlorinated inducers have been developed to modulate specific metabolic pathways or trigger defence mechanisms within the plant [6,24]. 3,5-Dichloroanthranilic acid (3,5-DCAA), a derivative of anthranilic acid, is a less investigated member of this group. It has shown potency in activating defence mechanisms in Arabidopsis [24] and more recently, also in barley (‘Hessekwa’ cv) [25]. However, effective priming and induced-resistance phenotypes are dependent on the plant genotype, and diverse cvs of the same crop can respond differently [5]. While the ‘Hessekwa’ cv exhibited susceptibility towards infection with *Pyrenophora teres* f. *teres,* the ‘Elim’ cv, developed for production as a winter rain-fed crop, exhibited greater resistance [23] and was chosen for further investigation. The main goal of this study was thus to apply metabolomic tools and approaches to the study of barley defence responses, specifically to evaluate the effect of the 3,5-DCAA on the metabolic landscape of the ‘Elim’ cv. If key compounds and metabolic pathways involved in the response can be revealed, this will lead to a better understanding of (i) the mechanism of action of 3,5-DCAA and (ii) the identification of potential biomarkers associated with the response.

## 2. Results

### 2.1. Visualisation of Reactive Oxygen Species (ROS) Production

**Figure 1:**
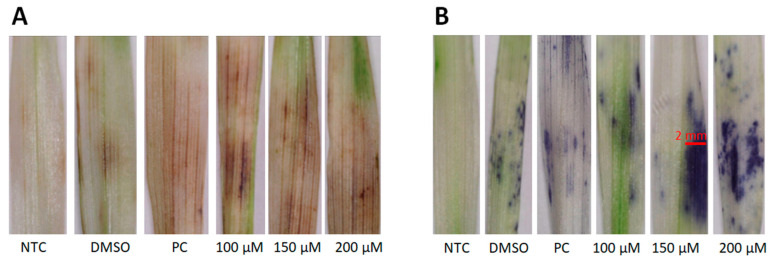
Qualitative determination of oxidative reactions in leaves of barley (*Hordeum vulgare*) treated with different concentrations of 3,5-dichloroanthranilic acid (100, 150, and 200 μM). (**A**) DAB stain for the determination of hydrogen peroxide and (**B**) NBT stain for the determination of superoxide radicals. NTC = nontreated controls, DMSO = solvent/vehicle control, PC = positive control, yeast cell wall elicitor, 100 µg mL^−1^.

The visual assessment of ROS production was completed with the histochemical stains diaminobenzidine (DAB) and nitro-blue tetrazolium (NBT). The principle behind the DAB stain relies on its oxidation by hydrogen peroxide (H_2_O_2_) in the presence of peroxidases to generate a dark brown precipitate [26,27]. NBT, on the other hand, reacts with superoxide anions (O_2_^•−^) to form a dark blue, insoluble formazan compound [28]. The formation of the colour products observed in Figure 1A,B revealed the in-situ presence of H_2_O_2_ and O_2_^•−^, respectively, in leaves pressure-infiltrated with 3,5-DCAA at all concentrations (100, 150, and 200 µM). Since ROS production is an early response that might trigger defence-related events [29], further experiments were conducted with 200 μM 3,5-DCAA.

### 2.2. LC-MS for Metabolite Separation and Detection

**Figure 2:**
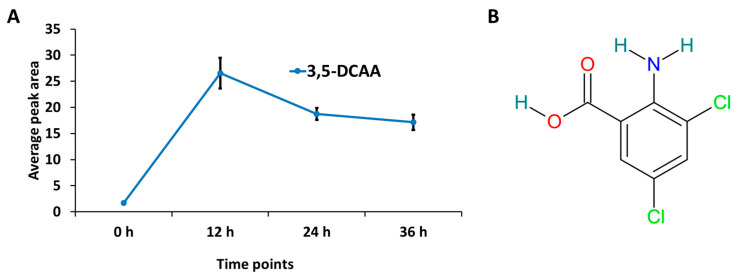
(**A**) Relative concentration of 3,5-DCAA in barley leaves following spray application of a 200 μM solution and incubation for 12, 24, and 36 h. Error bars indicate the standard deviations of the average peak areas of 3,5-DCAA present in the samples. (**B**) Structure of 3,5-dichloroanthranilic acid. Characteristic features of 3,5-DCAA-type inducers/elicitors are the backbone structure of a benzoic acid substituted with an amino group at position 2 and the presence of chlorines at positions 3 and 5.

Metabolomic studies employ advanced analytical techniques such as mass spectrometry (MS)-based analytical platforms, enabling the simultaneous identification and quantification of diverse metabolites. Here, an ultra-high-performance liquid chromatography (UHPLC)–high-definition MS analytical platform was used to unravel the metabolic fingerprints defining the 3,5-DCAA-induced states of young barley plants at different time points (12, 24, and 36 h post treatment). The presence of 3,5-DCAA was observed in the extracts at each of the time-points following spray application. The relative peak intensities corresponding to 3,5-DCAA were plotted in Figure 2A to illustrate the timeline in the plant after application. The initial uptake is evident in the 12 h extract, followed by a decrease that may correspond to the metabolism of the inducer at 24 and 36 h.

**Figure 3:**
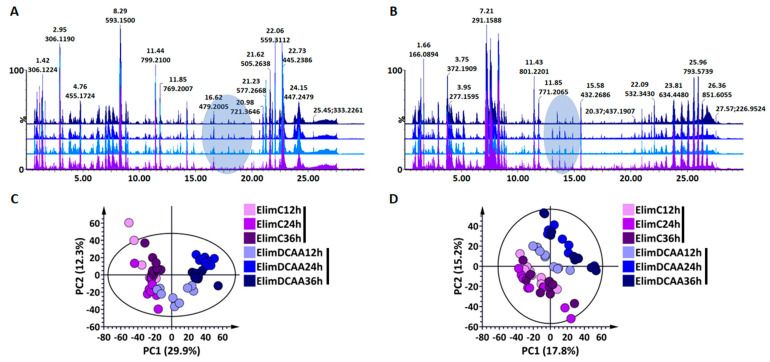
Base peak intensity (BPI) chromatograms generated by ultra-high-performance liquid chromatography—mass spectrometry (UHPLC–MS) in (**A**) negative and (**B**) positive ionisation modes for extracts of plants treated with 3,5-DCAA for 12, 24, and 36 h (light blue to dark blue, with purple representing the control of 24 h). The Y-axes are linked for comparison of relative peak intensities that are compared to the chromatogram of the control at 24 h (ElimC24h). The grey ellipses indicate some of the apparent differences at the BPI level. (**C**,**D**) show principal component analysis (PCA) score plots of ESI(–) and (+) data, respectively. All data were Pareto scaled, and the ellipses in each PCA score plot represent calculated Hoteling’s T^2^ with a 95% confidence interval. (**C**) A seven-component model explaining 69.1% variation and predicting 50.3% variation. (**D**) An eight-component model explaining 68.3% variation and predicting 43.0% variation.

The analysis of the UHPLC–MS chromatograms in both ionisation modes revealed treatment- and time-related differences, providing insights into the metabolic dynamics underlying the experimental treatments (Figure 3A,B illustrates the workflow that was followed). Although subtle, some quantitative and qualitative differences could be noted across a broad polarity range of compounds (e.g., as indicated by the grey shaded areas). In general, chromatographic data yield high-dimensional datasets with multiple variables, making it difficult to readily interpret and extract useful information. In addition to the visual chromatographic representation of the complex chemical profiles obtained, unsupervised chemometric modelling was performed to reduce the dimensionality of the datasets while revealing inherent patterns, structures, and relationships within the convoluted chemical profiles obtained from the samples. The generated principal component analysis (PCA) models show clear clusters, or groupings, separating treated and non-treated samples at each time point (Figure 3C,D).

**Figure 4:**
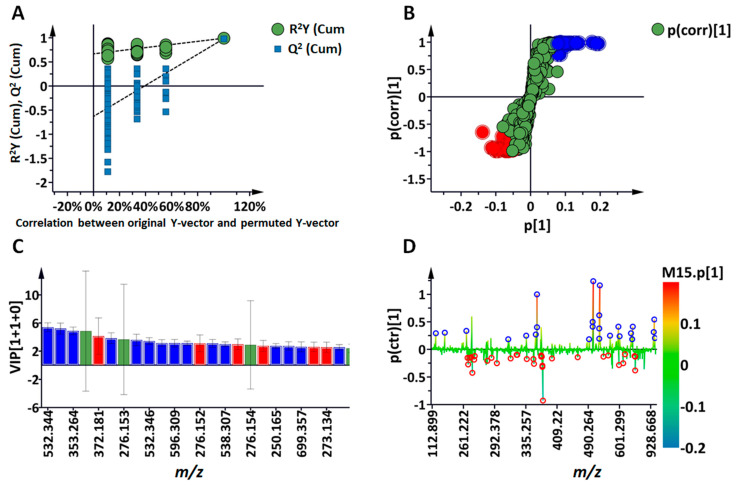
Orthogonal projection to latent structures discriminant analysis (OPLS-DA) for the selection of discriminant metabolites associated with the 3,5-DCAA treatment at 24 h. (**A**) shows a permutation test (*n* = 100) generated to evaluate the OPLS-DA model of ‘Control vs. 3,5-DCAA (*x*-axis, component 1 + 2 + 0; R^2^X = 0.502, R^2^Y = 0.991, Q^2^ = 0.980. CV-ANOVA = 6.65 × 10^−11^). For (**B**,**C**), the same colour code (red, blue, and green) was used, with (**B**) depicting the OPLS-DA loading S-plot showing selected features which are statistically significant in discriminating the two compared groups, control vs. 3,5-DCAA. Blue and red respectively indicate positive or negative correlation to the treatment, with green as unchanged. The reliability (correlation) and magnitude (covariance) of the samples in the model are shown on the axes as p(corr) and p [1], respectively. (**C**) Variable importance in projection (VIP) scores, >1 for each selected feature or *m*/*z* variable. (**D**) OPLS-DA S-lines highlight the differences between the treatment (in blue, positive values) and the control (in red, negative values) with regards to the occurrence of discriminant variables and their relative intensities.

To identify variables contributing to the clustering/groupings observed on the PCA score plots, a supervised learning method, orthogonal projection to latent structures discriminant analysis (OPLS-DA), was performed as exemplified in Figure 4, which shows the binary comparison of control vs. treatment at 24 h. Group separation was clearly observable in the OPLS-DA score space. A permutation test was used to assess the predictive capability of the generated OPLS-DA model (Figure 4A)**,** wherein the observed R^2^ and Q^2^ values were compared to the 100 randomly permutated ones. As evidenced in the example provided, the observed R^2^ and Q^2^ values of all computed models in this study outperformed their respective permuted counterparts. Using the OPLS loading S-plot (Figure 4B), variables positively or negatively correlated to the treatment were extracted at each time point. Only features exhibiting high correlation and covariation defined as [p(corr) ≥ 0.5, ≤−0.5, and (p1) ≥ 0.1, ≤−0.1] were considered. In addition, each selected feature was statistically evaluated using the variable importance in projection (VIP) score plots. A variable VIP score greater than 1 was used as a cut-off for selected features (Figure 4C). OPLS-DA S-lines were generated to better visualise the differences between sample classes (Figure 4D). In addition to the 24 h time point, OPLS-DA analyses were also performed for the 12 h and 36 h data sets against their respective controls. The list of annotated metabolites as compiled in Table 1 is thus a summary of the changes occurring in the metabolome over the time period 12–36 h.

**Figure 5:**
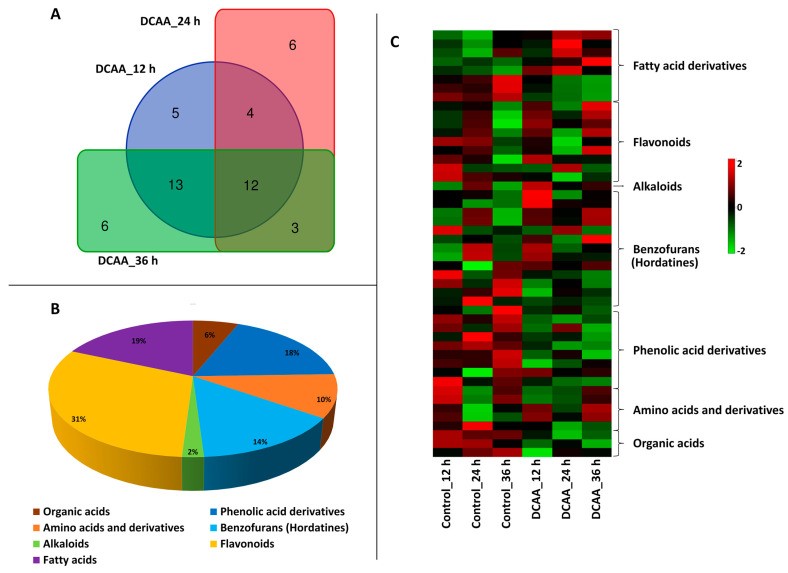
(**A**) Venn diagram of distribution of annotated metabolites across time points, showing specific and common metabolites. The details of these metabolites are found in the text and in Table 1. (**B**) Allocation of annotated metabolites classes and (**C**) heatmap showing the average levels of metabolites across all treatments (control 12, 24, and 36 h vs. DCAA 12, 24, and 36 h).

Metabolomic results are presented not merely as static snapshots but as dynamic portraits, capturing the flux and adaptability of cellular metabolism. The outcomes capture the interplay between metabolic pathways, unveiling potential biomarkers or metabolic signatures, and add novel insights into cellular responses. In total, 49 metabolites were putatively identified as contributing features to the sample groupings (Table 1). The Venn diagram generated in Figure 5A shows that five metabolites were specific to the 3,5-DCAA treatment at 12 h. These included the amino acids tyrosine and tryptophan, both negatively correlated to the treatment, the flavonoids isovitexin 7,6″-di-O-glucoside and apigenin 7-O-gentobioside (respectively up- and down-regulated to 3,5-DCAA), and a conjugate of 12-oxo-phytodienoic acid (OPDA). At 24 h, specific metabolites included N-acetylaspartylglutamic acid and feruloylagmatine isomer I (both negatively correlated to the treatment), as well as linolenoylglycerol isomer I, II, and a hordatine-related compound (all positively correlated to the treatment). Sinapoylagmatine, an isomer of hordatine A, isoorientin 7-O-[6″-feruloyl]-glucoside, a derivative of apigenin and of linolenic acid, and trihydroxyoctadecenoic acid (triHOME) were all specific to 36 h.

Twelve metabolites were common to all time points, and these were as follows: citric acid, *p*-coumaroylhydroxyagmatine, feruloylhydroxyagmatine, hordatine A hexose, hordatine B hexose, isovitexin, lutonarin (isoorientin-7-O-glucoside, a glycosyl-trihydroxyflavone), isovitexin 7-O-[6″-feruloyl]-glucoside, isoscoparin-7-O-glucoside, hydroxylinoleic acid, and linolenoylglycerol isomer III. Among the metabolites shared between 12 and 24 h, 3-feruloyquinic acid was positively correlated to both time points, and isovitexin 2″-O-glucoside was upregulated at 24 h. Saponarin, (also known as isovitexin-7-O-glucoside, a dihydroxyflavone), isoorientin 7-O-[6″-sinapoyl]-glucoside, apigenin derivative 1, and iso-scoparin-7-O-[6″-sinapoyl]-glucoside were found positively correlated to the treatment at 12 and 36 h. At 24 and 36 h, isoleucine was found positively correlated at both time points, and hordatine A isomer I and 9,12,13-triHODE isomer I were all negatively correlated to the 3,5-DCAA treatment.

These metabolites were dominated by the phenolic compounds, mainly flavonoids, representing about 31% of the annotated metabolites, Figure 5B. This was followed by fatty acid derivatives (19%), phenolic acid derivatives (18%) and benzofurans, and hordatines and derivatives (14%). Organic acids, amino acids and derivatives, and alkaloids made up 24% of all the annotated metabolites. All these metabolites were differentially distributed across time points as depicted in Figure 5C. The heatmap shows high and low accumulation across all treatments, representative of the metabolic reprogramming resulting from the 3,5-DCAA treatment. A positive correlation of metabolites with the treatment was mostly observed with fatty acid derivatives, flavonoids, and some benzofurans. Aromatic amino acids were found down-regulated at all time points. Among the fatty acid derivatives, while a linolenic acid derivative and linolenoylglycerol were up-regulated at all time points, hydroxylinolenic acid was down-regulated. Hordatine A and B hexose, hydroxytryptamine, and feruloylquinic acid were found fluctuating across time points. Among the flavonoids, saponarin was the most dominant and was found positively correlated to the treatment at all time points.

## 3. Discussion

Integrating metabolomics with investigations on induced resistance against plant diseases has opened new research and application avenues, highlighting its transformative potential for enhancing agricultural sustainability. The exogenous application of synthetic bioactive elicitors bypasses the initial recognition event and indirectly inhibits pathogen development by triggering the plant’s defence mechanisms. Chemical biology concerns the study of gene-to-metabolite functions in a cellular or organismal context using exogenous ligands, and the growing sophistication of this approach prompted its application to diverse biological processes [30]. The search for novel bioactive small molecules that produce a desired phenotype in plants is an established strategy in the agrochemical industry [14].

**Figure 6:**
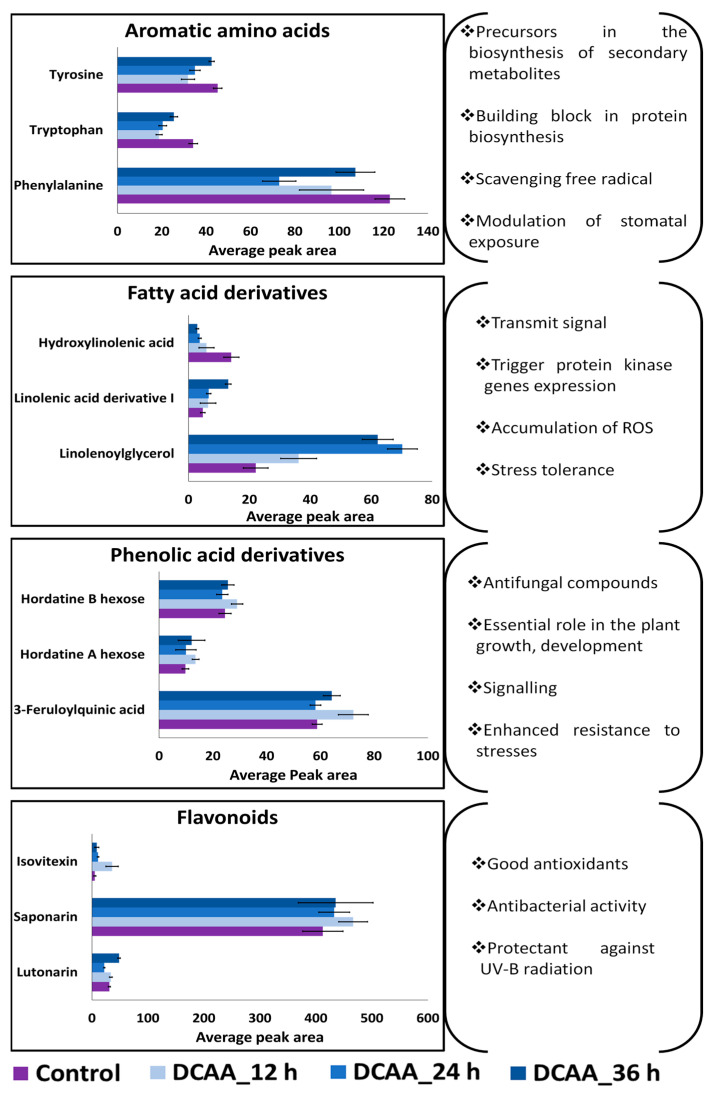
Relative quantification of selected metabolites and associated functions. These metabolites belong to the class of aromatic amino acids, fatty acid derivatives, phenolic acid derivatives, and flavonoids. The bar graphs are representative of the average of each metabolite peak area and give an indication of the abundance of the selected metabolite in the samples at 12, 24, and 36 h.

The discovery of more potent immune elicitors has been facilitated by the chemical modification of known plant molecules. Chemical biology can aid in understanding plant physiology, development, and defence mechanisms. With regards to the latter, one of the key areas of interest is the use of chemical inducers to manipulate plant responses. Here, 3,5-DCAA was applied to the leaves of the barley cv ‘Elim’ as an inducer of defence responses and possible priming agent. A summary of its effect on selected metabolites belonging to the designated metabolite classes is presented in Figure 6.

In a previous study on Arabidopsis, it was reported that dichlorinated molecules exhibit the strongest defence-inducing activity [31]. Loss of a single chlorine atom (especially in the three position) led to a loss or strongly reduced bioactivity, while the fully dechlorinated derivatives (i.e., anthranilic acid) were not active as inducers of defence [31]. Chlorinated aminobenzoates and hydroxybenzoates have the advantages of reduced phytotoxicity, increased stability, and prolonged activity [24]. In tobacco, via the induction and accumulation of pathogenesis-related (PR) proteins, mono- and dichlorinated compounds such as 3,5-dichlorosalicylic acid and 2,6-dichloroisonicotinic acid efficiently enhanced the plant’s resistance to pathogen attack [19,32,33]. The observed decrease in 3,5-DCAA over time in this study might be indicative of degradation or attempted detoxification of the dichlorinated elicitor. This might be accomplished by dehalogenase enzymes with the intracellular release of chlorine atoms that can potentially perturb the intracellular redox balance.

ROS production plays a role in stress signalling and plant redox-dependent immune responses, and the timing, extent, and location thereof is important [29,34]. The generation of ROS within the 3,5-DCAA-treated leaves, as shown by the histochemical stains indicating the presence of hydrogen peroxide and superoxide anion, is a novel finding and suggests the initiation of some oxidative events that might be similar to an oxidative burst that marks the beginning of early immune responses. Oxidative stress results from the imbalance between ROS generation and antioxidant defences and can trigger ROS-dependent signalling pathways or reset regulatory switches that transduce signals into responses, leading to adaptive changes in cellular biochemistry and homeostasis. Other responses resulting from the application of elicitors include the activation of phenylpropanoid and octadecanoid pathways and the production of phytoalexins, PR-proteins, and cell wall reinforcement polymers [35,36].

In this study, the activation of the pathways mentioned above was demonstrated by the fluctuation of pathway-related or pathway-specific metabolites (Table 1). Differential reprogramming can be displayed as high or low accumulation at specific time points, indicating early, late, or oscillatory responses [37]. The observed increasing or decreasing patterns can be associated with increased biosynthesis, involving interconversion reactions, conjugation, degradation, or incorporation into insoluble polymers such as lignin. Compounds identified as variable features or discriminatory metabolites include the amino acid phenylalanine that was negatively correlated to the treatment at 12 and 36 h. Phenylalanine is a key metabolite, serving as the central metabolic node connecting primary and secondary metabolisms in plants and feeding into the phenylpropanoid pathway [38,39]. In several studies, the activation of phenylalanine ammonia lyase (*PAL*) gene expression correlates with the induction of plant immune responses. In the presence of the PAL enzyme, phenylalanine is deaminated to *trans*-cinnamic acid, which is a precursor substrate in the formation of various hydroxycinnamic acid-intermediates, leading to the synthesis of phenylpropanoid metabolites [40,41,42]. Similarly, in some grasses, tyrosine can also be converted to 4-hydroxycinnamic acid in presence of tyrosine ammonia lyase (TAL) [42]. In addition to phenylalanine, tyrosine and tryptophan were also annotated as discriminant metabolites, showing negative correlation with the 3,5-DCAA treatment at all time points. The decreased concentrations of these aromatic amino acids may be associated with their utilisation for the accumulation of defence-related secondary metabolites such as phenolic acid derivatives, flavonoids, and alkaloids [39,43]. Such build-ups included hydroxytryptamine, 3-feruloyquinic acid, lutonarin, isovitexin 7,6″-di-O-glucoside, saponarin, isoscoparin-7-O-glucoside, isovitexin, isovitexin, isoorientin 7-O-[6″-feruloyl]-glucoside, isoscoparin-7-O-[6″-sinapoyl]-glucoside, isovitexin 7-O-[6″-feruloyl]-glucoside, and two apigenin derivatives. These compounds are known to play essential roles in plant growth, development, signalling, pigmentation, and defence against biotic and abiotic stresses [44,45].

**Figure 7:**
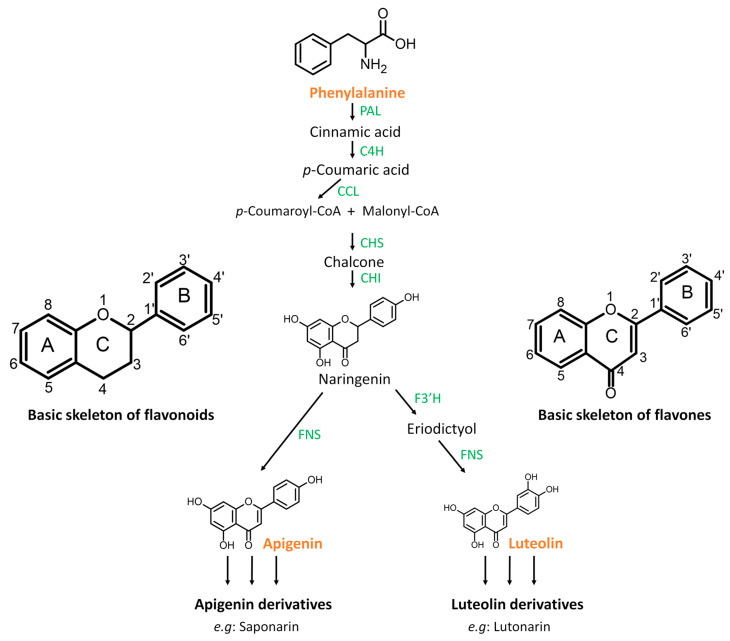
Simplified representation of multi-step enzymatic reactions of apigenin and luteolin biosynthesis. PAL: phenylalanine ammonia lyase; C4H: cinnamate 4-hydroxylase; CCL: coumaroyl-CoA ligase; CHS: chalcone synthase; CHI: chalcone isomerase; F3′H: flavone 3′-hydroxylase; FNS: flavone synthase. The structures on the sides of the central pathway represent the core (skeleton) structure of flavonoids and flavones. FNS directs flavanones to the flavone route. Flavones are flavonoids characterised by a double bond between C-2 and C-3. The sugar residues of known natural flavonoid C-glycoside compounds are primarily attached to the C-6 and C-8 of the A ring or as 7-O-glycosides to the hydroxyl group on C-7. In addition, hydroxycinnamic acids may be attached as sinapoyl and feruloyl units to the 6” position of the sugar residues.

As an example of a pathway deriving from the phenylpropanoid pathway, a summary of the biosynthesis of flavonoids, more specifically flavones, is illustrated in Figure 7. Flavonoids are frequently depicted as C6-C3-C6 compounds. Depending on the chemical structure, degree of oxidation, and unsaturation of the C3 linking chain, flavonoids can be classified into different sub-groups, such as flavanones, flavonols, flavones, isoflavones, chalcones, and anthocyanins.

Although increased flux into the phenylpropanoid pathway in response to 3,5-DCAA is apparent, it is noteworthy that only flavones (flavonoids containing a 2-phenyl-1-benzopyran-4-one backbone) derived from apigenin or luteolin, products of the enzyme flavone synthase (FNS) [46], were annotated as discriminant features. As flavonones, they are known for their ability to scavenge free radicals [47,48]. Their antioxidant and antimicrobial properties [49] contribute to increasing the plant’s resilience and ability to withstand pathogen attack.

Such reactions often required energy, as evidenced by the implication of the tricarboxylic acid cycle (TCA), indicated by the down-regulation of citric and isocitric acids, as well as malic acid. The TCA cycle is a central metabolic pathway, playing a critical role in cellular respiration by generating energy through the oxidation of acetyl-CoA derived from carbohydrates, lipids, and proteins. The observed decrease in the levels of these organic acids reflects an increase in metabolic activity and energy demand to meet the biosynthetic and bioenergetic needs. Modifications in the levels of carboxylic acids were observed in plants during stress responses, leading to the hypothesis that tricarboxylates could also modulate signal transduction cascades linked to plant defence responses [50,51,52].

Membrane lipids are the source of octadecanoid fatty acids linked to the octadecanoid pathway. Phospholipases are involved in plant resistance by maintaining fatty acid pools for jasmonic acid (JA) synthesis and activating the JA signalling pathway. Here, signature metabolites included an OPDA conjugate as well as derivatives of linolenic and linoleic acids. These polyunsaturated fatty acids are substrates of lipoxygenase (LOX) and serve as precursor compounds in signalling cascades triggering defence mechanisms against wounding and pathogen attacks [53]. The end-products and intermediates resulting from LOX mechanisms (such as OPDA) can transmit signals and trigger the protein kinase gene expression which result in ROS accumulation and participate in the development of stress tolerance [54,55,56,57]. The occurrence of conjugates and derivatives of these compounds can be attributed to the plant’s state of alertness instead of that of active defence.

Since induced resistance phenotypes are frequently dependent on the genotype, diverse cultivated varieties of the same species can exhibit different responses [5]. We previously reported on a related study on the barley cv, ‘Hessekwa’ [25], susceptible to net-blotch disease caused by *Pyrenophora teres* f. *teres* [23]. The untargeted metabolomics analysis using principal component analysis and hierarchical cluster analysis indicated that non-stimulated ‘Hessekwa’ and ‘Elim’ differ significantly at a metabolome level [58]. Nonetheless, a broadly similar observation was made regarding their responses to 3,5-DCAA, pointing to the ability of the inducer to redirect metabolic resources towards e.g., the phenylpropanoid pathway and the activation of key metabolites in defence mechanisms or immune responses in both cvs [18].

During plant stress responses, the phenylpropanoid pathway frequently collaborates with the polyamine pathway and in the case of the ‘Hessekwa’ cv, we reported that a synchronised increase in the relative content of hordatines as well as precursors of hordatines, highlights the role of both the pathways in plant immunity [18]. The role of these barley-specific metabolites in antimicrobial defences was previously highlighted [59], and the increase in their production might be related to the preconditioning of the seedlings. In contrast, although identified as discriminant biomarkers, the profile of hordatines in the ‘Elim’ cv (this study) did not show a continuous increase over the 36 h of investigation. This observation might be interpreted that the hodatines may function more as pre-existing phytoanticipins than as inducible phytoalexins in ‘Elim’.

Other comparable observations made with ’Hessekwa’ include the decrease in the aromatic amino acids which correlated with the biosynthesis of downstream secondary metabolites [18]. Another observation was the positive correlation of linolenoylglycerol isomers to 3,5-DCAA, particularly at 36 h post-treatment. With regards to flavonoids, the flavone lutonarin was up-regulated at all time points while saponarin was only positively correlated to the treatment at 24 h. Although presenting a similar pattern of response in terms of presence or absence of most discriminant metabolites, the two cvs seemingly present nuanced differences in their responses to 3,5-DCAA. In this context, it should be considered that metabolomic complexity arises not only due to chemical diversity, but also due to varying concentrations and the dynamic range of the metabolites. In addition, in the case of inducible responses, the timing, duration and extent of such metabolic changes might differ and thus play a significant role in contributing to metabolomic differences/variability. This may contribute to their differential phenotypes (susceptibility or resistance to future challenges) and reiterates the necessity of conducting complementary or comparative studies to elucidate the interaction of a species or cultivars of the same species with an elicitor or the environment.

Our results support the concept that reprogramming of the plant metabolome is a crucial adaptive mechanism to cope with stressful events and one of the defensive strategies employed by plants [60,61]. When metabolic changes last beyond the recovery from stress events, it constitutes a metabolic imprint, or altered state of metabolism [34,62]. Similarly, priming can be regarded as an imprint that functions to prepare for future stresses, so as to integrate preceding histories of diverse environmental stresses [63,64]. These authors further proposed that metabolic imprints may be used as a memory that stores and processes information as a central component of plant priming [64,65].

The treatment of barley leaves with 3,5-DCAA resulted in reconfiguration of the metabolome at different time-points. Discriminant metabolites were grouped as amino acids and derivatives, organic acids, phenolic acid derivatives, benzofurans, flavonoids, and alkaloids. The changes observed in these metabolite classes can be explained as being in support of plant defence mechanisms or the state of alertness. The negative correlation of the aromatic amino acids (phenylalanine, tyrosine, and tryptophan) with the treatment is associated with the biosynthesis of defence-related secondary metabolites. Both the phenylpropanoid and the octadecanoid pathways were major routes activated by 3,5-DCAA treatment. A review of present and past results on 3,5-DCAA seems to suggest a role as both an inducer of defence responses as well as a priming agent, as defined by [66].

Besides their possible uses for disease control and management in plant pathosystems, synthetic defence elicitors may also serve as investigative agents and adaptable/versatile tools for chemical genetic analyses of plant defence mechanisms and ligand-based discovery of gene function. By dissecting the biochemical interactions and cellular responses triggered during priming, a forward genetics approach can point to the structural or regulatory genes associated with the new phenotype. Future studies should address the in planta metabolic fate of 3,5-DCAA, identifying its biochemical targets and generating details on the interaction (modulation or perturbation) with these targets. From this, researchers can develop innovative strategies to bolster crop protection, improve yield, and ensure sustainable agricultural practices in the face of increasing environmental challenges.

## 4. Materials and Methods

### 4.1. Barley Cultivation and Preparation of 3,5-DCAA Solutions

Seeds from the barley cv ‘Elim’ (a rain-fed, winter-cultivated malting variety) were provided by the South African Barley Breeding Institute (SABBI, Bredasdorp, Western Cape, South Africa). Seeds were surface-sterilised with 70% ethanol and soaked in sterile water for 2 h prior to sowing in soil (Germination mix, Culterra, Muldersdrift, South Africa), then pasteurised at 70 °C. An average of 20 seeds were sown in each pot (three pots per treatment), measuring 8 cm in depth and 12 cm in diameter. The watering of the plants was performed twice a week with water and a solution containing a water-soluble chemical fertiliser (Multisol, Culterra, Muldersdrift, South Africa). Seedlings were kept in a regulated growth environment with a 12 h light–dark cycle at 22 to 27 °C until 16 d post-emergence or 21 d after planting, corresponding to physiological stage 13 according to the Zadoks growth and development scale [67]. The priming inducer 3,5-dichloroanthranilic acid (3,5-DCAA) was purchased from Merck-Sigma-Aldrich, (Johannesburg, South Africa). 3,5-DCAA was dissolved in dimethylsulphoxide (DMSO, 1 μL mL^−1^; Merck-Sigma-Aldrich, (Johannesburg, South Africa) and mixed with 0.05% wetting agent (Effekto, Pretoria, South Africa) in distilled water to obtain the desired concentrations. The experimental plan included three independent biological replicates.

### 4.2. Histochemical Evaluation of ROS Production

The 3,3-diaminobenzidine (DAB; 1 mg mL^−1^) and nitro-blue tetrazolium chloride (NBT; 2 mg mL^−1^) solutions were freshly made with acidified water (pH 3.6) and phosphate-buffered saline, respectively. The leaf segments, pressure-infiltrated with 100, 150, and 200 µM of 3,5-DCAA, were stained in 10 mL of DAB or NBT solution and incubated overnight. The negative control (NTC) was not pressure-infiltrated, the positive control (PC) was pressure-infiltrated with a cell wall-derived elicitor from yeast (100 µg mL^−1^) [68], and DMSO (1 μL mL^−1^) was used as a vehicle control. The chlorophyll was then removed with boiling ethanol (96%) [27,28].

### 4.3. Treatment with 3,5-Dichloroanthranilic Acid, Metabolite Extraction, and Sample Preparation for Liquid Chromatography–Mass Spectrometry Analysis (LC–MS)

Approximately 6 mL (40 sprays) of 200 μM 3,5-DCAA was applied on the leaves of the seedlings, while the controls received only the DMSO solution. The leaf material was harvested at 12, 24, and 36 h post-treatment and snap-frozen in liquid nitrogen to quench metabolic activity. Samples were stored in −80 °C for later use. Metabolites were extracted as previously described [25]. Briefly, the leaves were ground with liquid nitrogen, then 80% cold methanol was added (1:10 *w*/*v* ratio) and the mixture further homogenised using an Ultra-Turrax homogeniser. Subsequently, all samples were centrifuged, and the hydromethanolic supernatants were evaporated to complete dryness to concentrate the extracts. The residues were finally dissolved in 50% methanol (a tenth of the original volume), filtered, and prepared for LC–MS analysis.

### 4.4. Mass Spectrometry-Based Sample Analyses: Ultra-High-Performance Liquid Chromatography–High-Definition Mass Spectrometry (UHPLC–HDMS)

The analysis of the aqueous methanol extracts was performed with a Waters Acquity UHPLC coupled to a Waters SYNAPT G1 QTOF (quadrupole time-of-flight) high-definition mass spectrometer system (Waters Corporation, Milford, MA, USA). The HSS T3 C18 column (150 mm × 2.1 mm × 1.8 µm, Waters Corporation) was thermostatted at 60 °C and used for the reverse-phase chromatographic separation of extracts. The mobile phase consisted of mass spectrometry-grade water and acetonitrile (Romil, Cambridge, UK) and formic acid (Sigma-Aldrich, Merck, Johannesburg, South Africa). Eluents A (water) and B (acetonitrile), both containing 0.1% formic acid, were used for the concave gradient elution, running at a flow rate of 0.4 mL min^−1^. The elution commenced with 5% B for the first min and gradually increased to 95% B over 24 min. The chromatographic conditions were then adjusted to 10% A and 90% B for 10 s, followed by 5% A and 95% B for 1 min 50 s before restoration to the initial conditions for column equilibration for 2 min. The injection volume was 2 µL, and the total run time was 30 min. To account for analytical variability and to prevent measurement bias, each sample was analysed in triplicate. The sample order was randomised, and blanks consisting of 50% methanol were injected to monitor the background noise, possible sample carry-over, and solvent contamination. The stability of the LC-MS system was monitored by inserting quality control (QC) samples in the batches. Data acquisition involved three independent biological replicates, with each replicate analysed in triplicate, resulting in a total sample size of *n* = 9 as required for multivariate data analyses.

The high-resolution, accurate mass TOF-MS analyser was used in a V-optics mode, with the centroid spectral data acquired in both negative and positive electrospray ionisation (ESI) modes. Operating parameters included masses ranging from 50 to 1200 Da and a scan time of 0.1 s; the capillary voltage set at 2.5 kV; and sampling and extraction cone voltages at 40 V and 4.0 V, respectively. The desolvation and cone gas flows were set at 550 L h^−1^ and 50 L h^−1^, respectively, with nitrogen used as the nebulisation gas at a flow rate of 700 L h^−1^. A desolvation temperature of 450 °C and a fixed source temperature of 120 °C were used. Leucine encephalin ([M − H]^−^ = 554.2615 and [M + H]^+^ = 556.2766) at a concentration of 50 pg mL^−1^ served as the reference mass calibrant and was sampled every 15 sec to generate an average intensity of 350 counts per scan. This reference helped the processing software (MassLynx XS^TM^ 4.1, Waters Corporation, Milford, MA, USA) to perform automatic correction of slight centroid mass deviations observed in the samples, ensuring precise mass measurements with typical mass accuracy ranging from 1 to 3 mDa. Both intact and fragmented data were acquired using an MS^E^ method with collision energies ranging from 10 to 40 eV. The fragmentation data were employed for subsequent metabolite structural elucidation and annotation.

### 4.5. Data Processing, Data Mining, and Metabolite Annotation of Discriminant Metabolites

The application manager MarkerLynx-XS within the MassLynx-XS 4.1 software (Waters, Manchester, UK) was used for data pre-processing. This software generates a matrix with mass spectra information corresponding to each variable/feature and including *m*/*z* pairs, retention time (Rt), and normalised and integrated peak areas. The pre-processing parameters were set as follows: Rt range = 0.6–27 min (ESI+) and 0.6–23 min (ESI−), mass range = 50–1200 Da, mass tolerance = 0.05 Da, and the intensity threshold = 50 counts. The data matrices considered for subsequent chemometric and statistical analyses were those with noise level below the MarkerLynx cut-off of 50%.

For chemometric analysis, the resulting data matrices were imported into the Soft Independent Modelling of Class Analogy (SIMCA) software, version 14 (Sartorius, Umeå, Sweden). The chemometric models generated included principal component analysis (PCA), an unsupervised method which provided an explorative overview of the data and indicated patterns and groupings corresponding to the time-dependent metabolic changes in response to the treatment. For biological characterisation and interpretation of the clusters/profiles observed on the PCA models, a supervised method, Orthogonal Projection to Latent Structures-Discriminant Analysis (OPLS-DA), was generated. The OPLS-DA models allowed sample classification and the identification of features associated with different groupings. The OPLS-DA models were validated with permutation tests where the statistical parameters of the generated models were compared with 100 permutated ones. In addition, a seven-fold cross-validation (CV) was also evaluated (CV-ANOVA *p* < 0.05). OPLS-DA-generated loadings, or ’S-plots’, were evaluated for variable selection. Features with high reliability (correlation) and influence (covariation) (p(corr) ≥ 0.5, ≤−0.5 and (p1) ≥ 0.1, ≤−0.1) were considered, while variable importance in projection (VIP) plots were utilised to assess the statistical significance of each feature (cut-off > 1) and prevent potential bias during feature selection. The S-lines/plots were also generated to illustrate the abundance of each metabolite across the two compared treatments. The S-lines appear like longitudinal oscillation, and the relative abundance of each feature is determined by a colour code ranging from blue to red (from less abundant to more abundant, respectively).

### 4.6. Metabolite Annotation and Heatmap Analyses

The annotation and putative identification of analytes identified as discriminatory metabolites were conducted in accordance with the Metabolomics Standards Initiative (MSI) level 2 guidelines [69]. This relied on MS data, which included the accurate mass as well as the mass-fragmentation patterns. The molecular formula of a selected ion representing a metabolite feature was determined through manual searches using bioinformatics tools and databases such as the Dictionary of Natural Products (accessed on 24 January 2024) [70], ChemSpider (accessed on 22 January 2024) [71], and PubChem (accessed on 27 February 2024) [72]. Fragmentation patterns in the mass spectra, generated at increasing collision energies, were scrutinised and validated by comparing them with existing data on barley and related plant species found in the scientific literature [73,74,75]. As an additional data visualisation tool, the relative distribution of annotated metabolites across the time points were visualised on 2-dimensional dendrogram heatmaps generated on the MetaboAnalyst 6.0 (accessed on 28 April 2024) [76].

## Figures and Tables

**Table 1:** List of all identified discriminant metabolites selected from the OPLS-DA S-plots. The generated data were derived from the barley cv ‘Elim’, treated with 3,5-DCAA, and harvested after incubation periods of 12, 24, and 36 h. For each time point, the treatment was compared to its corresponding control. ‘Up’ and ‘down’ indicate positive and negative association or correlation to the treatment. Metabolites were annotated in agreement with level 2 of the Metabolomics Standards Initiative (MSI).

No	ESI *	Compound	Rt (min)	Mass (*m*/*z*)	12 h	24 h	36 h
**Organic acids**							
1	Neg	Isocitric acid	0.91	191.0170	Down		Down
2	Neg	Malic acid	0.96	133.0113	Down		Down
3	Neg	Citric acid	1.14	191.0168	Down	Down	Down
**Alkaloids**							
4	Pos	Hydroxytryptamine	1.67	177.1035		Up	
**Amino acids and derivatives**							
5	Pos	Isoleucine	1.29	132.1110		Up	Up
6	Pos	Phenylalanine	1.65	166.0867	Down		Down
7	Pos	Tryptophan	2.42	205.0966	Down		
8	Pos	Tyrosine	2.43	188.0697	Down		
9	Neg	N-Acetylaspartylglutamic acid	5.78	303.0885		Down	
**Fatty acids**							
10	Neg	Linolenic acid derivative I	14.27	675.2672			Up
11	Neg	9,12,13-TriHODE	16.55	327.2148		Down	Down
12	Neg	TriHOME	17.29	329.2307			Down
13	Neg	OPDA conjugate	20.00	309.2041	Down		
14	Pos	Linolenoylglycerol isomer I	21.01	353.2680		Up	
15	Pos	Linolenoylglycerol isomer II	21.86	353.2656		Up	
16	Pos	Linolenoylglycerol isomer III	22.10	353.2641	Up	Up	Up
17	Neg	Hydroxylinoleic acid	22.25	295.2254	Down		Down
18	Neg	Linolenic acid derivative II	22.73	445.2349		Up	
**Phenolic acid derivatives**							
19	Neg	Coumaric acid derivative	0.86	404.1007	Down		Down
20	Neg	3-Feruloyquinic acid	3.91	367.1000	Up	Up	
21	Neg	Sinapic acid hexose	5.16	385.1110	Down		Down
22	Pos	*p*-Coumaroylhydroxyagmatine	2.54	293.1536	Down	Down	Down
23	Pos	Feruloylagmatine isomer I	3.11	307.1771		Down	
24	Pos	Feruloylhydroxyagmatine	3.35	323.1702	Down	Down	Down
25	Pos	Coumaroylagmatine	3.99	277.1609	Down		Down
26	Pos	Feruloylagmatine isomer II	5.21	307.1715	Down		Down
27	Pos	Sinapoylagmatine	6.07	337.1863			Down
**Benzofurans (Hordatines)**							
28	Pos	Hordatine B hexose	3.80	743.3729	Up	Down	Up
29	Pos	Hordatine related compound	3.89	177.0551		Up	
30	Pos	Hordatine A hexose	4.14	713.3628	Up	Down	Up
31	Pos	Hordatine A isomer I	5.83	551.3078		Down	Down
32	Pos	Hordatine B isomer I	7.30	291.1585	Down		Down
33	Neg	Hordatine A isomer II	7.68	595.2989			Down
34	Pos	Hordatine B isomer II	7.88	291.1548	Down		Down
**Flavonoids**							
35	Pos	Isoorientin-7-O-glucoside/Lutonarin	6.40	611.1589	Up	Down	Up
36	Neg	Isovitexin 7,6″-di-O-glucoside	8.17	755.2106	Up		
37	Pos	Isovitexin-7-O-glucoside/Saponarin	8.31	595.1665	Up		Up
38	Pos	Isovitexin-7-O-rhamnosyl-glucoside	8.81	741.2220	Down	Down	
39	Pos	Isoscoparin-7-O-glucoside	8.97	625.1743	Up	Down	Up
40	Pos	Isovitexin 2″-O-glucoside	9.81	595.1663	Down	Up	
41	Pos	Apigenin 7-O-gentobioside	9.95	565.1560	Down		
42	Neg	Isoorientin 7-O-[6″-sinapoyl]-glucoside	10.54	815.2071	Up		Up
43	Pos	Isovitexin	10.60	433.1110	Up	Up	Up
44	Pos	Isoorientin 7-O-[6″-feruloyl]-glucoside	10.87	787.2114			Up
45	Neg	Isovitexin-7-O-[6″-sinapoyl]-glucoside	11.44	799.2127	Down	Down	
46	Neg	Isoscoparin-7-O-[6″-sinapoyl]-glucoside	11.55	829.2247	Up		Up
47	Neg	Isovitexin 7-O-[6″-feruloyl]-glucoside	11.85	769.2014	Up	Down	Up
48	Neg	Apigenin derivative 1	11.92	665.2814	Up		Up
49	Neg	Apigenin derivative 2	12.04	665.2501			Up

* ESI: Electrospray ionisation, positive or negative mode; OPDA: 12-oxophytodienoic acid.

## Data Availability

Raw data, data processing information, and the meta-data have been deposited into the National Metabolomics Data Repository (NMDR) website, the Metabolomics Workbench, https://www.metabolomicsworkbench.org, where it has been assigned Study ID ST-003399 (Release date 31 August 2024). PR002105. doi: 10.21228/M8HV52.
